# HDAC5, an early osimertinib-responsive gene, is a novel therapeutic target for the drug resistance in *EGFR*-mutant lung adenocarcinoma cells

**DOI:** 10.1016/j.bbrep.2025.102016

**Published:** 2025-04-15

**Authors:** Hanbing Lyu, Akihiko Ishimura, Ryusuke Suzuki, Khurelsukh Buyanbat, Gerelsuren Batbayar, Makiko Meguro-Horike, Shin-ichi Horike, Seiji Yano, Takeshi Suzuki

**Affiliations:** a Division of Functional Genomics, Cancer Research Institute, Kanazawa University, Kanazawa, Japan; b Laboratory of Molecular Biology, Institute of Biology, Mongolian Academy of Sciences, Mongolia; c Division of Integrated Omics Research, Research Center for Experimental Modeling of Human Disease, Kanazawa University, Kanazawa, Japan; d Department of Respiratory Medicine, Faculty of Medicine, Institute of Medical, Pharmaceutical, and Health Sciences, Kanazawa University, Kanazawa, Japan; e Nano Life Science Institute, Kanazawa University, Kanazawa, Japan

**Keywords:** HDAC5, LMK235, Lung cancer, EGFR-TKI, Osimertinib, Drug resistance

## Abstract

Aberrant epigenetic regulation is closely associated with drug tolerance, an early step in the acquisition of drug resistance. We previously reported that a pioneer transcriptional factor (also called an epigenetic initiator) rapidly induced by osimertinib, a third-generation epidermal growth factor receptor (EGFR) tyrosine kinase inhibitor, plays a pivotal role in promoting the formation of osimertinib-tolerant cells. In this study, to identify novel epigenetic factors associated with osimertinib-tolerance, we performed a comprehensive screening of epigenetic factors whose expression is rapidly induced by osimertinib. Our results revealed that *HDAC5*, a class IIa histone deacetylase (HDAC), is a prominently induced epigenetic regulator in several *EGFR*-mutant non-small cell lung cancer (NSCLC) cell lines during the early response to osimertinib. Knockdown of *HDAC5* significantly reduced the emergence of osimertinib-resistant cells. Furthermore, treatment with LMK235, a selective HDAC5 inhibitor, significantly increased global histone acetylation and enhanced osimertinib-induced apoptosis. These findings highlight the potential of HDAC5 as a novel therapeutic target to overcome osimertinib-resistance and suggest LMK235 as a promising compound to provide therapeutic benefit to *EGFR*-mutant NSCLC patients receiving osimertinib treatment.

## Highlights

• *HDAC5*, a class IIa HDAC, is an early response gene to osimertinib.
• *HDAC5* knockdown increased the osimertinib-sensitivity of *EGFR*-mutant cancer cells.
• LMK235, a selective HDAC5 inhibitor, is superior in combination with osimertinib.
• LMK235 increased histone H3 acetylation and enhanced osimertinib-induced apoptosis.

## 1 Introduction

Lung cancer is the leading cause of cancer-related death worldwide [1]. Non-small cell lung cancer (NSCLC) accounts for 85 % of cases and often harbors epidermal growth factor receptor (*EGFR*) mutations [2]. While EGFR tyrosine kinase inhibitors (EGFR-TKIs) initially provide significant therapeutic benefits to patients with *EGFR*-mutations, they inevitably experience recurrence within a few years due to the emergence of drug-resistant tumors. Osimertinib (ADZ9291), a third-generation EGFR-TKI, irreversibly inhibits constitutively active EGFR mutants, including exon 19 deletion (del19), L858R, and T790 M mutations, without affecting wild-type EGFR [3]. However, like other EGFR-TKIs, the efficacy of osimertinib is still limited by the acquisition of drug resistance through both EGFR-dependent mechanisms (e.g., C797S, a secondary *EGFR* mutation) and EGFR-independent mechanisms, such as the activation of bypass signaling pathways (e.g., *MET* amplification) or histological transformations. Therefore, many investigators have explored combination approaches targeting these acquired phenotypes to circumvent this resistance [3].

We recently demonstrated that FOXA1, a pioneer transcriptional factor, functions as an epigenetic initiator by activating the expression of insulin-like growth factor-1 receptor (*IGF-1R*) [4]. This activation leads to the emergence of osimertinib-tolerant cells, the precursors of drug-resistant cells. Unlike acquired drug resistance, drug tolerance is reversible, highlighting the critical role of epigenetic regulation in this process [3]. Aberrant post-translational modifications of histones, including histone methylation, are closely associated with the emergence of drug tolerant cells, known as drug tolerant persisters (DTPs) [5]. Notably, a global reduction in histone acetylation has been observed in DTPs, and treatment with trichostatin A, a pan-HDAC inhibitor, eliminates DTPs [5]. Thus, reduced histone acetylation is considered a hallmark of early drug resistance, and restoring acetylation may represent a promising therapeutic strategy to overcome it. FOXA1, which we identified, and other DTP factors like AXL [6] have been reported to be immediately upregulated by osimertinib. To better understand the molecular mechanism governing the early stages of drug resistance, it is important to identify epigenetic factors immediately induced by osimertinib and to analyze their roles.

In this study, we performed a microarray screening and identified *HDAC5*, a class IIa HDAC, which was rapidly upregulated by osimertinib in *EGFR*-mutant lung cancer cells. We demonstrated that treatment of LMK235, a selective inhibitor of HDAC5, could dramatically increase the efficacy of osimertinib, proposing a novel combination therapy to overcome osimertinib resistance.

## 2 Materials and methods

### 2.1 Plasmids and cell culture

Lentiviral vectors (pLKO.1-puro, Sigma) expressing small hairpin RNAs (shRNAs) targeting scramble (Scr, Sigma) and *HDAC5* were prepared as previously described [7]. The sequences of oligonucleotides for *HDAC5*-specific shRNAs (sh1 and sh5) are shown in Table S1.

This study used three human non-small cell lung cancer (NSCLC) cell lines harboring *EGFR* mutations. HCC827 and H1975 cells were purchased from the American Type Culture Collection (ATCC), and PC9 cells were obtained from the RIKEN BRC Cell Bank (Japan).

### 2.2 Quantitative reverse transcribed PCR (qRT-PCR) analysis

qRT-PCR analysis was conducted with ThunderBird SYBR qPCR Mix (Toyobo) on ViiA7 real-time system (Applied systems). *GAPDH* expression was used as a control for normalization. The primer's sequences for qRT-PCR are provided in Table S1. Each PCR assay was carried out in quadruplicate.

### 2.3 Western blotting analysis

Western blotting was conducted according to the protocol described previously [8]. Each quantification was normalized with TBP expression, and the averages from three independent experiments are presented with standard deviations.

### 2.4 Cell proliferation assay

HCC827 cells (4x10^3^) were treated with osimertinib (Selleckchem) at concentrations ranging from 0 to 20 nM, or DMSO (dimethyl sulfoxide; Wako) was used as a control. After 96 h of treatment, cell proliferation was assessed using the Cell Counting Kit-8 (CCK-8; DOJINDO Lab., Japan) in accordance with the manufacturer's instructions. Measurements were performed using four independent wells per sample.

### 2.5 Colony formation assay

HCC827 cells were treated with 5 nM osimertinib and 50 nM LMK235 (Selleckchem) for 19 days. For H1975 and PC9 cells, treatments consisted of either 100 nM osimertinib and 200 nM LMK235, or 100 nM osimertinib plus 50 nM LMK235, with culture durations of 12 days and 9 days, respectively. All experiments were carried out in triplicate.

### 2.6 Flow cytometry

Cells were pre-treated with 100 nM LMK235 for three days, followed by exposure to 100 nM LMK235, 5 nM osimertinib, or their combination. After 24 h of treatment, the cells were harvested, fixed, and analyzed by flow cytometry. Each cell cycle analysis was performed in triplicate.

### 2.7 Statistical analysis

A two-tailed student's t-test was applied to assess the statistical significance of the difference between the control and the samples. ∗∗∗, *P* < 0.001; ∗∗, *P* < 0.01; ∗, *P* < 0.05; n.s., not significant.

### 2.8 Microarray analysis

Microarray was conducted following a previously described protocol [9]. The microarray data have been deposited in the Gene Expression Omnibus (GEO) database, under accession number GSE283536.

Detailed descriptions are shown in Supplementary Materials and Methods.

## 3 Results

### 3.1 *HDAC5* is an epigenetic factor relating to early osimertinib-response in *EGFR*-mutant lung cancer cells

Previously, we found that FOXA1, a pioneer transcriptional factor (also called an epigenetic initiator), induced *IGF-1R* expression upon treatment with osimertinib, a third-generation EGFR-TKI. The resulting increase in IGF-1R provides compensatory survival signaling in place of EGFR, leading to the emergence of drug-tolerant cells [4]. Since osimertinib could rapidly upregulate the expression of *FOXA1* within 12 h, we hypothesize that early epigenetic regulation might be crucial for acquiring osimertinib resistance. To identify novel epigenetic factors induced by osimertinib, we first performed microarray analysis using total RNA from HCC827 cells (harboring the delR746-A750 EGFR mutation) treated with 50 nM osimertinib for 6 h. As shown in Fig. S1A, we compared expression profiles between osimertinib-treated and control cells, and identified 811 genes whose expression was more than 2-fold higher in treated cells. Using the public database, Epifactors [10], we selected 911 protein-coding genes of epigenetic factors and cross-referenced them with our upregulated gene list. This analysis yielded 16 candidate epigenetic factors. Notably, *TET1* was identified, a gene reported as an EGFR-TKI-induced factor in lung cancers and glioblastomas [11], supporting the reliability of our approach. Next, we performed qRT-PCR to validate whether these candidate genes were consistently upregulated more than 2-fold in several *EGFR*-mutant lung cancer cell lines treated with osimertinib (Fig. S1B). In HCC827 cells, 9 out of 16 candidate genes were shown to be induced by osimertinib. We further screened the candidates showing more than 2-fold upregulation in other lung cancer cell lines, H1975 (L858R and T790 M *EGFR* mutation) and PC9 (delR746-A750 EGFR mutation) cells treated with 100 nM osimertinib, and identified *HDAC5*, one of histone deacetylases (Figs. S1C, S1D, 1B, and 1C). Even at a lower concentration of osimertinib (5 nM), *HDAC5* was significantly induced in HCC827 cells within 12 h (Fig. 1A). Importantly, HDAC5 protein levels were consistently elevated across all three *EGFR*-mutant cell lines post-treatment (Fig. 1D). These findings suggest that *HDAC5* plays a conserved and functionally significant role in early cellular response to osimertinib.

**Fig. 1:**
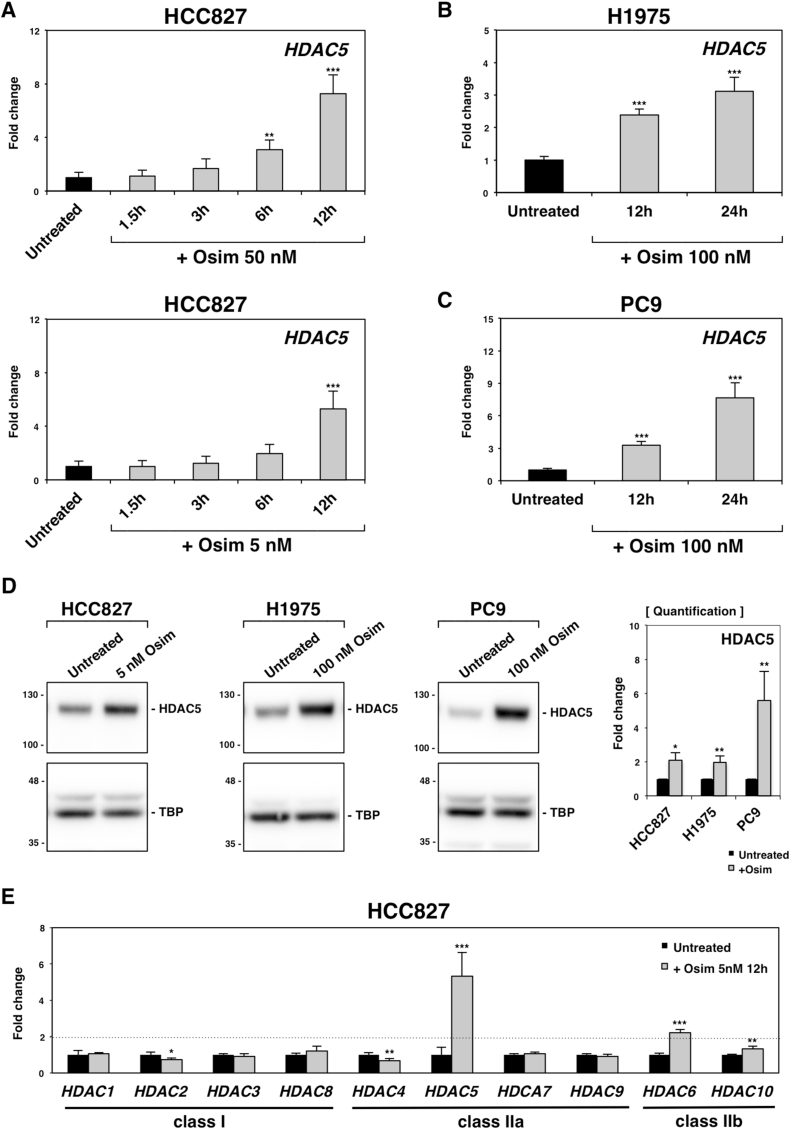
*HDAC5* expression was upregulated by osimertinib in *EGFR* mutant lung cancer cells. (A) Treatment with 50 nM (top) or 5 nM (bottom) osimertinib caused the significant upregulation of *HDAC5* in HCC827 cells within 12 h. (B, C) *HDAC5* was induced in H1975 (B) and PC9 (C) cells following 12 h of treatment with 100 nM osimertinib. (D) HDAC5 protein and TBP (loading control) were detected in HCC827, H1975, and PC9 cells ± osimertinib by immunoblotting. Band quantification is shown on the right. (E) The expression of class I and class II HDACs was analyzed in HCC827 cells treated with 5 nM osimertinib for 12 h ∗∗∗*P* < 0.00.1, ∗∗*P* < 0.01, and ∗*P* < 0.05, as analyzed by Student's t-test.

*HDAC5* belongs to the class IIa family of histone deacetylases, and we investigated whether other class IIa (*HDAC4*, *HDAC7*, and *HDAC9*) and IIb (*HDAC6* and *HDAC10*) family members respond to osimertinib. We also examined the expression of class I histone deacetylases (*HDAC1*, *HDAC2*, *HDAC3*, and *HDAC8*), which play a central role on histone deacetylation in nucleus [12]. The results showed that *HDAC5* and *HDAC6* were significantly up-regulated (>2-fold) in osimertinib-treated HCC827 cells (Fig. 1E), whereas only *HDAC5* reached this level of induction in H1975 and PC9 cells (Fig. S2). Therefore, we focused on HDAC5 as a key member of the HDAC family associated with early osimertinib response.

### 3.2 Depletion of *HDAC5* in HCC827 cells reduced the cell viability in the presence of osimertinib

Since previous reports demonstrated oncogenic roles of *HDAC5* in various cancers such as breast, liver, colon, and lung cancer [13], we hypothesized that inhibition of *HDAC5* induction might affect the cellular tolerance to osimertinib. To prove our hypothesis, we prepared *HDAC5* knockdown HCC827 cells using two different *HDAC5*-specific shRNAs (sh1 and sh5) and confirmed through western blotting that both effectively suppressed endogenous HDAC5 protein levels (Fig. 2A). Then, we used the CCK-8 assay to compare the survival rates of knockdown cells and control cells across various concentrations of osimertinib (Fig. 2B). The results showed a significant decrease in survival rates for the knockdown cells at osimertinib concentrations of 2.5–10 nM compared to the controls. Additionally, a colony formation assay was performed to evaluate cell viability after prolonged osimertinib treatment (Fig. 2C), revealing a remarkable reduction of resistant colonies in the knockdown cells. These findings suggest that *HDAC5* depletion enhances the efficacy of osimertinib, highlighting HDAC5 as a potential therapeutic target for overcoming drug resistance.

**Fig. 2:**
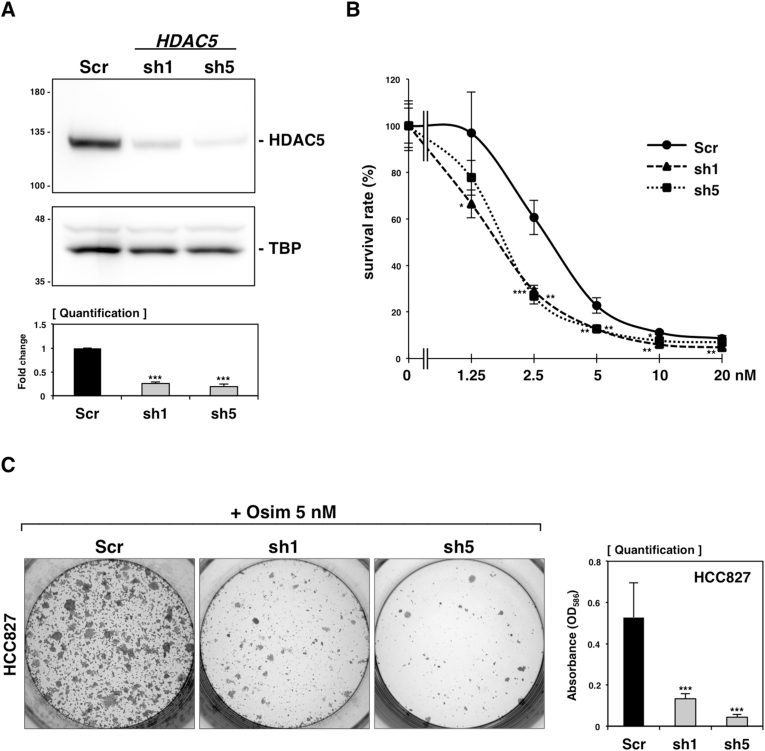
Knockdown of *HDAC5* in HCC827 cells significantly reduced the viability of HCC827 cells treated with osimertinib. (A) Western blotting indicated the knockdown efficiency of *HDAC5*-specific shRNAs (sh1 and sh5). Scramble (Scr) shRNA was used as a negative control. Band quantification is displayed below the corresponding images. (B) The CCK-8 assay assessed the viability of scramble and *HDAC5* knockdown cells across a range of osimertinib (0–20 nM). (C) Colony formation assays were conducted with scramble and *HDAC5* knockdown cells treated with 5 nM osimertinib. After two weeks, the colonies were stained by crystal violet. Subsequently, the absorbance at 586 nm was measured for quantification (right). ∗∗∗*P* < 0.00.1, ∗∗*P* < 0.01, and ∗*P* < 0.05, as analyzed by Student's t-test.

### 3.3 The combination of LMK235, an HDAC5 selective inhibitor, with osimertinib significantly reduced drug-resistant colonies in *EGFR*-mutant lung cancer cell lines

To evaluate whether an HDAC5-specific inhibitor could produce a similar combined effect with osimertinib, we treated HCC827 cells with 50 nM LMK235 [14], a small molecular inhibitor against HDAC5, in combination with 5 nM osimertinib (Fig. 3A). Consistent with the knockdown experiments (Fig. 2), the colony formation assay revealed that this combination significantly reduced cell viability compared to osimertinib alone. Additional assays were conducted using H1975 and PC9 cells (Fig. 3B and C), where 200 nM of LMK235 with 100 nM osimertinib or 50 nM of LMK235 with 100 nM osimertinib were applied, respectively. The combination treatment markedly reduced the number of drug-resistant colonies in both cell lines. In contrast, administrating LMK235 alone did not affect cell proliferation in any of the tested cell lines (Fig. S3). Thus, these results strongly indicate that LMK235 is a promising drug candidate for combination therapy with osimertinib.

**Fig. 3:**
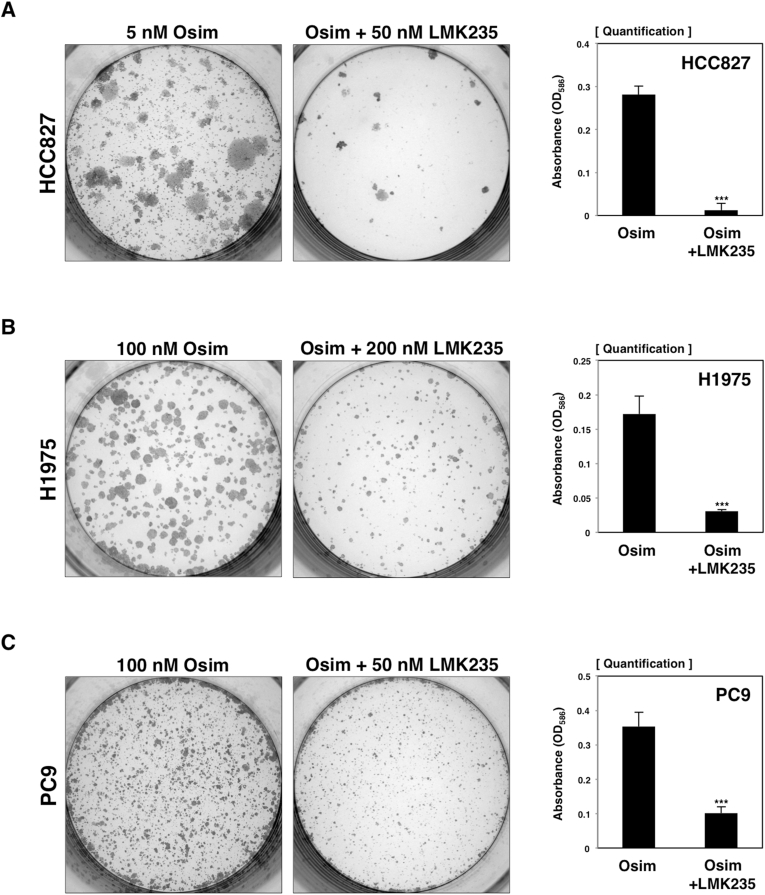
The combination of osimertinib with LMK235, a selective HDAC5 inhibitor, strikingly decreased the colony-forming activity of HCC827 (A), H1975 (B), and PC9 (C) cells. Drug names and concentrations are indicated above each panel. Graphs on the right show quantification from colorimetric analyses. ∗∗∗*P* < 0.00.1, as analyzed by Student's t-test.

### 3.4 Treatment of LMK235 combined with osimertinib led to apoptosis in the treated cells possibly through a high level of histone H3 acetylation

We next investigated how LMK235 enhanced the efficacy of osimertinib. Since LMK235 was developed based on its cytotoxicity and enzymatic inhibitory activity against histone deacetylases [14], we assessed its impact on global histone H3 acetylation (H3K9/27ac). To inhibit HDAC5-dependent epigenetic regulation, HCC827 cells were pre-treated with 100 nM LMK235 for 72 h, followed by an additional 24-h treatment with 100 nM LMK235, 5 nM osimertinib, or their combination. Western blot analysis showed increased levels of histone H3 acetylation in LMK235-treated cells, regardless of osimertinib co-treatment (Fig. 4A). In contrast, quantitative analysis of Fig. 4A revealed that LMK235 did not affect endogenous HDAC5 protein levels or its induction by osimertinib in HCC827 cells (Fig. S4). These finding suggest that LMK235 imodulates the enzymatic activity, and thereby the function, of HDAC5 rather than its expression. Additionally, we evaluated EGFR activation by measuring phosphorylated EGFR proteins (Fig. 4A). The results clearly showed that osimertinib abolished EGFR phosphorylation, confirming its effectiveness in this study.

**Fig. 4:**
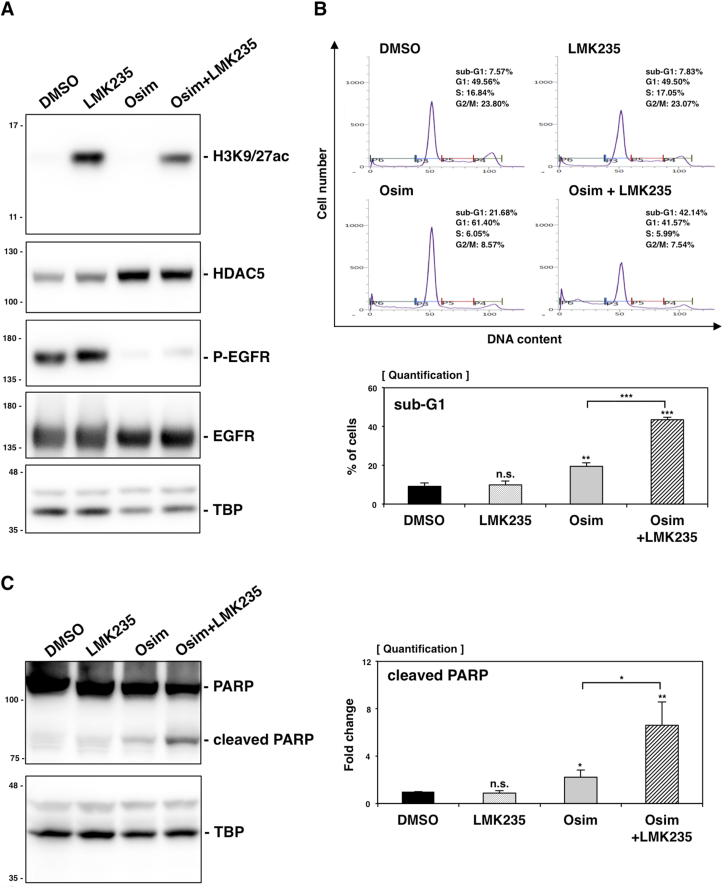
The combination of LMK235 and osimertinib efficiently promoted apoptosis in HCC827 cells. (A) HCC827 cells were treated for 24 h with DMSO (vehicle), 50 nM LMK235, 5 nM osimertinib, or their combination. Protein lysates were analyzed by western blotting to detect acetylated Histone H3 (H3K9/27ac), HDAC5, phosphorylated EGFR (P-EGFR), or total EGFR (pan-EGFR), and TBP. (B) Flow cytometry (top) revealed cell cycle distribution in HCC827 cells treated with LMK235, osimertinib, or their combination. The sub-G1 population (%) for each group -DMSO (black bar), LMK235 (punctate bar), osimertinib (grey bar), and their combination (striped bar)- is summarized in the statistical analysis (bottom). (C) Cleaved PARP, an apoptotic marker, was detected by immunoblotting in HCC827 cells treated with LMK235, osimertinib or both. Quantification of the bands is shown on the right. ∗∗∗*P* < 0.00.1, ∗∗*P* < 0.01, and ∗*P* < 0.05, as analyzed by Student's t-test. n.s., not significant.

To evaluate whether the combination of LMK235 and osimertinib effectively induced cell death in HCC827 cells, we performed cell cycle analysis using flow cytometry on cells treated with LMK235, osimertinib or both (Fig. 4B). The analysis revealed a significantly increased sub-G1 phase population in osimertinib-treated cells (21.68 %) compared to DMSO controls (7.57 %), while LMK235-treated cells (7.83 %) displayed no notable difference from the controls. Notably, the combination treatment significantly increased sub-G1 phase (42.14 %) than osimertinib alone, indicating a synergistic promotion of apoptosis. We also examined cleaved Poly-ADP-ribose polymerase (PARP), one of the early markers of apoptosis [15], by western blotting (Fig. 4C). The combination treatment significantly increased cleaved PARP levels compared to either drug alone. In contrast, treatment with LMK235 alone did not alter the cell cycle distribution or produce cleaved PARP under our experimental conditions. Altogether, these results strongly suggest that LMK235 amplifies osimertinib-induced apoptosis, highlighting the co-treatment as a promising strategy to overcome osimertinib resistance in *EGFR*-mutant lung cancer cells.

## 4 Discussion

In this study, we found that the expression of *HDAC5* was immediately upregulated by osimertinib and that silencing *HDAC5* significantly suppressed the emergence of osimertinib-resistant cells. Furthermore, the selective HDAC5 inhibitor LMK235 enhanced the efficacy of osimertinib, with the combination treatment markedly reducing the number of resistant colonies. Mechanically, LMK235 was found to induce a global increase in histone acetylation, thereby sensitizing cancer cells to osimertinib-induced apoptosis. Collectively, these findings identify HDAC5 as a novel therapeutic target for overcoming osimertinib-resistance and highlight LMK235 as a promising candidate for combination therapy.

HDAC5 is overexpressed in various cancers, where its aberrant regulation contributes to malignant phenotypes such as increased cell proliferation, evasion of apoptosis, enhanced invasion, metastasis, and drug resistance [13]. Depletion of *HDAC5* enhanced sensitivity to chemotherapeutic agents, doxorubicin and cisplatin, in MCF7 and HeLa cells [16]. Moreover, HDAC5 was implicated in resistance to sorafenib, a multi-tyrosine kinase inhibitor, in hepatocellular carcinoma cells [17]. These findings highlight the potential involvement of HDAC5 in acquiring drug resistance. However, its role in osimertinib resistance in NSCLC had not been previously investigated. Thus, this study provides the first evidence identifying HDAC5 as a novel therapeutic target to overcome osimertinib resistance.

Aberrant upregulation of HDAC genes is common in both solid and hematological tumors and closely linked to cancer development [18]. While HDAC inhibitors show efficacy in hematologic malignancies, their success in solid tumors is limited [18]. Preclinical studies suggest that combining HDAC inhibitors with chemotherapy or molecular-targeted drugs, including EGFR-TKIs, may improve therapeutic outcomes. EGFR-TKIs (e.g., gefitinib, osimertinib) combined with pan-HDAC inhibitors (e.g., trichostatin A, vorinostat) enhance growth inhibition and synergistically reduce the viability of *EGFR*-mutant lung cancer cells through apoptosis [2,5]. Isoform-selective HDAC inhibitors, such as HDAC3 and HDAC6 inhibitors, also exhibit synergy with EGFR-TKIs, highlighting their therapeutic potential [19,20]. Consistent with these findings, we used LMK235, an HDAC5-selective inhibitor, which shows promise in overcoming osimertinib-resistance. LMK235 was originally synthesized to inhibit both HDAC5 and HDAC4 [14], and thus, we cannot exclude the possibility that its anti-cancer effects involve inhibition of endogenous HDAC4 activity. However, osimertinib specifically upregulated HDAC5, not HDAC4 (Fig. 1E). Therefore, the combinatory effects of LMK235 with osimertinib may reflect preferential inhibition of HDAC5, the isoform upregulated by osimertinib. Previous reports demonstrated that combining HDAC inhibitors with EGFR-TKIs synergistically reduced the viability of cell lines that have already acquired drug resistance [2]. Whether LMK235 exerts similar effects on such resistant cell lines remains unknown. Therefore, further investigation using resistant cell lines and animal models is essential to validate the therapeutic potential of this HDAC inhibitor in these contexts.

We previously reported that osimertinib induces drug tolerance by upregulating *IGF-1R* gene expression via the pioneer transcription factor FOXA1 and that this upregulation is associated with increased local histone acetylation at the *IGF-1R* locus [4]. These findings appear to be paradoxical in light of the present study, where the HDAC5 inhibitor LMK235 reduced drug resistance through global histone acetylation enhancement (Fig. 4A). To address this discrepancy, we examined whether LMK235 affects the expression of *IGF-1R* and *FOXA1* in HCC827 cells. The observed upregulation was modest, with *IGF-1R* and *FOXA1* increasing only 1.4-fold and 1.5-fold, respectively (Fig. S5). Despite this limited induction, LMK235 treatment significantly reduced osimertinib resistance. These findings suggest that specific target genes regulated by HDAC5 enzymatic activity might play a critical role in drug resistance. We plan to identify these key target genes in the future.

Structural and biochemical analyses have long suggested that class IIa HDACs, including HDAC5, exhibit significantly lower deacetylase activity compared to class I HDACs [21]. However, recent research indicates that the catalytic domain of HDAC5 is critical for NRF2-dependent antioxidant gene expression, which plays a role in reducing oxidative stress in cardiomyocyte [22]. In this study, we used LMK235, a selective inhibitor of class IIa catalytic activity, and found clear evidence that LMK235 increased histone acetylation (Fig. 4A) and significantly suppressed colony formation when combined with osimertinib (Fig. 3). These results are consistent with the previous report indicating that restoring histone acetylation contributes to overcoming drug resistance [5]. Our findings suggest a functional link between HDAC5 catalytic activity and epigenetic regulation. Nonetheless, the relationship between HDAC5's deacetylase activity and its biological functions remains a subject of debate [22], warranting further investigation.

In summary, our findings suggest that combination therapy targeting HDAC5 may offer significant benefits for *EGFR*-mutant lung cancer patients treated with osimertinib. A comprehensive understanding of the molecular mechanism of HDAC5 will be essential for developing more effective and advanced therapeutic strategies in the future.

## CRediT authorship contribution statement

**Hanbing Lyu:** Validation, Investigation. **Akihiko Ishimura:** Writing – original draft, Visualization, Validation, Methodology, Investigation, Funding acquisition, Conceptualization. **Ryusuke Suzuki:** Investigation, Data curation. **Khurelsukh Buyanbat:** Investigation. **Gerelsuren Batbayar:** Investigation. **Makiko Meguro Horike:** Investigation, Data curation. **Shin-ichi Horike:** Investigation, Data curation. **Seiji Yano:** Resources, Investigation. **Takeshi Suzuki:** Writing – review & editing, Supervision, Project administration, Funding acquisition, Conceptualization.

## Funding

This work was supported in part by Grants-in-Aid for Scientific Research C (grant numbers: 22K07146 to A. I., 22K06880 to T. S.) from the Ministry of Education, Culture, Sports, Science and Technology of Japan. This work was also supported by MEXT Promotion of Development of a Joint Usage/Research System Project: Coalition of Universities for Research Excellence Program (CURE) Grant Number JPMXP1323015484.

## Declaration of competing interest

Seiji Yano, one of our co-authors, has received collaborative research funding from Chugai Pharmaceutical (Japan) and Eli Lilly (United States), as well as honoraria for his lecture from Chugai, AstraZeneca (United Kingdom), and Eli Lilly, which may constitute potential competing interests. All other authors declare that they have no conflicts of interest in relation to this article.

## Figures and Tables

**figs1:**
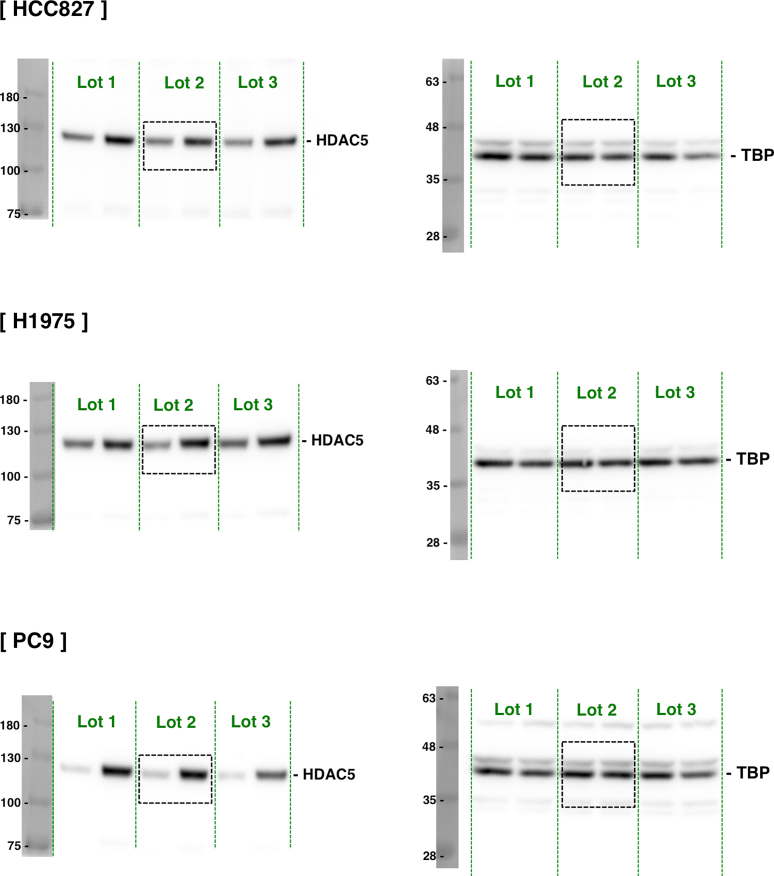


**figs2:**
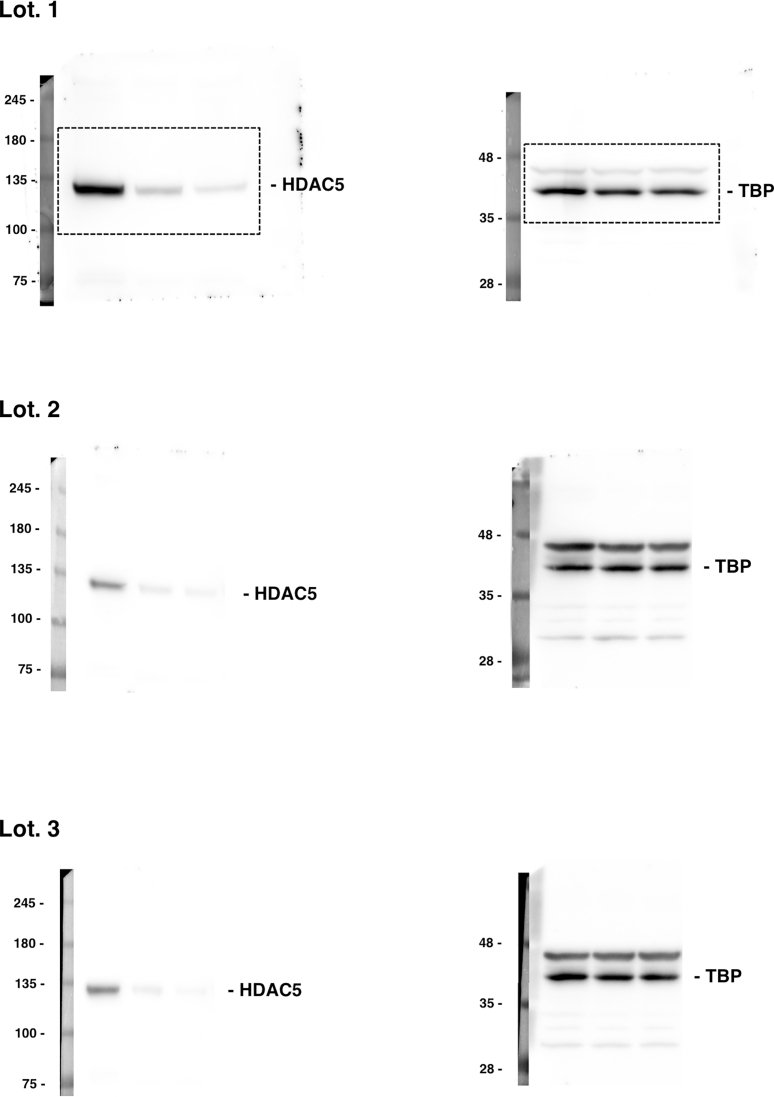


**figs3:**
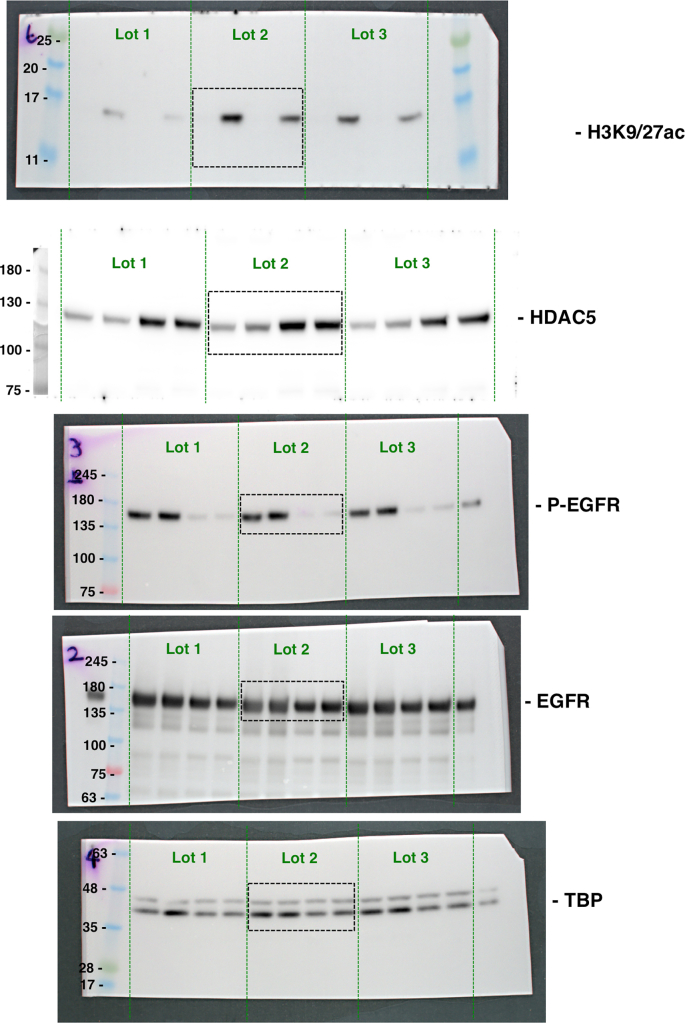


**figs4:**
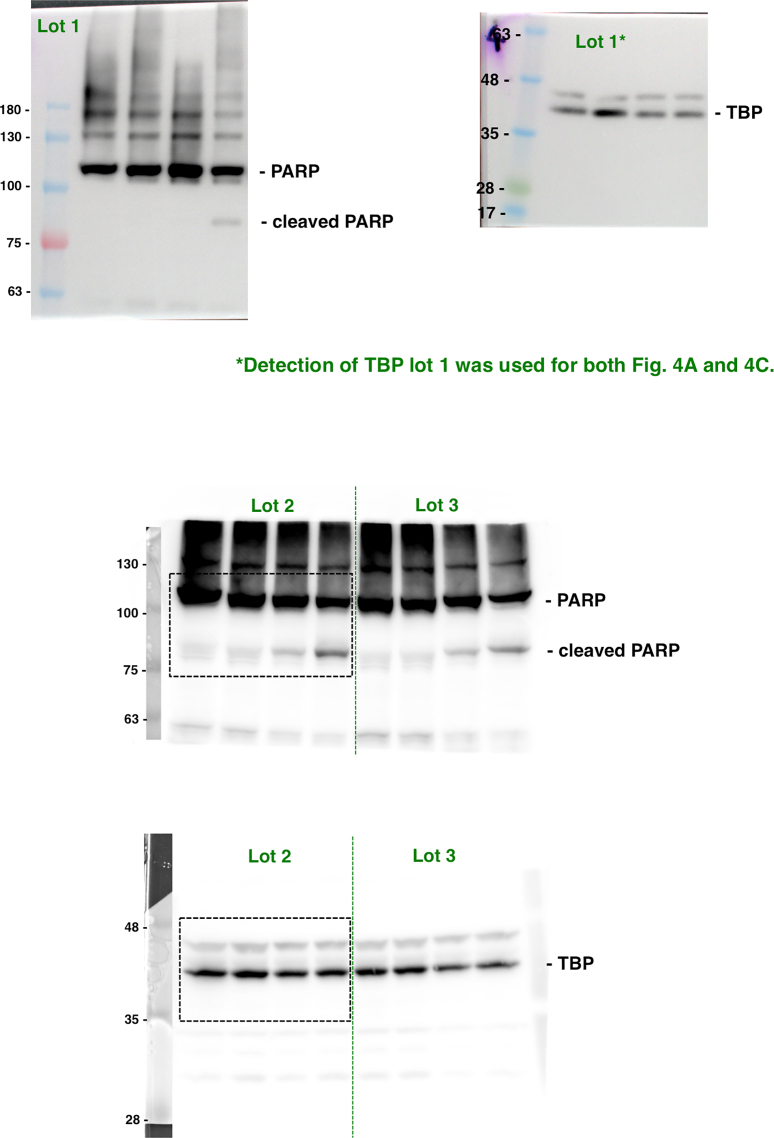


## Data Availability

Data will be made available on request.
