# Supplementary material for: HDAC5, an early osimertinib-responsive gene, is a novel therapeutic target for the drug resistance in EGFR-mutant lung adenocarcinoma cells
Source: Biochem Biophys Rep. 2025 Apr 15;42:102016. doi: 10.1016/j.bbrep.2025.102016 (PMC12022642; doi:10.1016/j.bbrep.2025.102016)
Supplement: Multimedia component 1 [file mmc1.docx]

**Supplementary Materials and Methods**

2.1 Plasmids and cell culture

Lentiviral vectors (pLKO.1-puro, Sigma) expressing small hairpin RNAs (shRNAs) targeting scramble (Scr, Sigma) and *HDAC5* were prepared as previously described [1]. The sequences of oligonucleotides for *HDAC5*-specific shRNAs (sh1 and sh5) were shown in Table S1.

This study used three human non-small cell lung cancer (NSCLC) cell lines harboring *EGFR* mutations. HCC827 cells were purchased from the American Type Culture Collection (ATCC) and cultured in DMEM (Nacalai) medium. H1975 cells were a generous gift from Dr. Yano (Kanazawa Univ., Japan), and PC9 cells were obtained from the RIKEN BRC Cell Bank (Japan). H1975 and PC9 cells were maintained in RPMI-1640 (Wako) medium. All culture medium contained 10% fetal bovine serum (FBS), L-glutamine and penicillin/streptomycin (Wako). The absence of mycoplasma contamination in the cell lines was confirmed using the MycoAlert Mycoplasma Detection Kit (Lonza).

2.2. Quantitative reverse transcribed PCR (qRT-PCR) analysis

Total RNA was extracted with the RNA-iso Plus (Takara) following to the manufacturer’s protocol and then reverse transcribed into cDNA using the Superscript VILO cDNA synthesis kit (Invitrogen). qRT-PCR analysis was conducted with the ThunderBird SYBR qPCR Mix (Toyobo) on the ViiA7 real-time system (Applied systems). *GAPDH* expression was used as a control for normalization. The primer’s sequences for qRT-PCR are provided in Table S1. Each PCR assay was carried out in triplicate.

2.3 Western blotting analysis

Western blotting was conducted according to the protocol described previously [2]. For detecting phosphorylated EGFR protein, the Phosphatase Inhibitor Cocktail, EDTA-free (Nakarai) was supplied in the sample lysates, and 5 % bovine serum albumin (Wako) was used as a blocking reagent for immunoblotting. To detect acetylated histone H3, protein samples were separated on the SuperSep Ace 15-20 % tricine gel (Wako). Antibodies used in this study were described below; anti-HDAC5 (D1J7V, Cell Signaling Technology), anti-TBP (Proteintech), anti-EGFR (D38B1, Cell Signaling Technology), anti-phosphorylated EGFR (D7A5, Cell Signaling Technology), and anti-H3K9/27ac (MABI0310 Monoclonal Inst. Japan). Band intensities were quantified using the Fusion Software (Vilber Lourmat). Each quantification was normalized with TBP expression, and the averages from three independent experiments are presented with standard deviations.

2.4 Cell proliferation assay

HCC827 cells (4x10^3^) were plated in 96-well plated (Thermo). The next day, osimertinib (Selleckchem) was added to the culture medium at concentrations ranging from 0 to 20 nM, or DMSO (dimethyl sulfoxide; Wako) was used as a control. The medium was replaced every three days. After 96 hours of treatment, cell proliferation was assessed using the Cell Counting Kit-8 (CCK-8; DOJINDO Lab., Japan) in accordance with the manufacturer’s instructions. Measurements were performed using four independent wells per sample.

2.5 Colony formation assay

HCC827 (5x10^4^) H1975 (1x10^3^), and PC9 (5x10^3^) cells were seeded into 6-well plated (Corning). HCC827 cells were treated with 5 nM osimertinib and 50 nM LMK235 (Selleckchem) for 19 days. For H1975 and PC9 cells, treatments consisted of either 100 nM osimertinib and 200 nM LMK235, or 100 nM osimertinib plus 50 nM LMK235, with culture durations of 12 days and 9 days, respectively. All experiments were carried out in triplicate. Crystal violet staining and quantification were conducted according to a previous report [3].

2.6 Flow cytometry

Cells were pre-treated with 100 nM LMK235 for three days, followed by exposure to 100 nM LMK235, 5 nM osimertinib, or their combination. After 24 hours of treatment, the cells were harvested and fixed in ice-cold 70% ethanol. The fixed cells were washed with 1xPBS, incubated with RNase A (Sigma), and stained with propidium iodide (Wako) prior to analysis. Flow cytometric analysis was performed using BD FACSLyric (Beckton-Dickinson), and the proportion of cells in each phase of the cell cycle was determined using FACSuite (Beckton-Dickinson). Each cell cycle analysis was performed in triplicate.

2.7 Statistical analysis

A two-tailed student’s t-test was applied to assess the statistical significance of the difference between the control and the samples. ***, *P* < 0.001; **, *P* < 0.01; *, *P* < 0.05; n.s., not significant.

2.8 Microarray analysis

Microarray was conducted following a previously described protocol [4]. Total RNA was isolated from HCC827 cells expressing control shRNA (Scr) treated with or without 50 nM osimertinib for 6 hours. Cyanine-3 (Cy3)-labeled cRNAs were hybridized to SurePrint G3 Unrestricted GE 8x60K (G4858A #72363, Agilent Technologies). The scanned images were analyzed with Feature Extraction Software 11.0.1.1 (Agilent) using default parameters. Data were normalized and filtered with three filters with GeneSpring software 14.9 (Agilent Technologies). The microarray data have been deposited in the Gene Expression Omnibus (GEO) database, under accession number GSE283536.

**Supplementary** **References**

[1] Z. Enkhbaatar, M. Terashima, D. Oktyabri, S. Tange, A. Ishimura, S. Yano, T. Suzuki, KDM5B histone demethylase controls epithelial-mesenchymal transition of cancer cells by regulating the expression of the microRNA-200 family, Cell Cycle 12 (2013) 2100-2112. 10.4161/cc.25142.

[2] A. Ishimura, K. Minehata, M. Terashima, G. Kondoh, T. Hara, T. Suzuki, Jmjd5, an H3K36me2 histone demethylase, modulates embryonic cell proliferation through the regulation of Cdkn1a expression, Development 139 (2012) 749-759. 10.1242/dev.074138.

[3] B. Bonnekoh, A. Wevers, F. Jugert, H. Merk, G. Mahrle, Colorimetric growth assay for epidermal cell cultures by their crystal violet binding capacity, Arch Dermatol Res 281 (1989) 487-490. 10.1007/BF00510085.

[4] G. Batbayar, A. Ishimura, H. Lyu, S. Wanna-Udom, M. Meguro-Horike, M. Terashima, S.I. Horike, T. Takino, T. Suzuki, ASH2L, a COMPASS core subunit, is involved in the cell invasion and migration of triple-negative breast cancer cells through the epigenetic control of histone H3 lysine 4 methylation, Biochem Biophys Res Commun 669 (2023) 19-29. 10.1016/j.bbrc.2023.05.061.

**Supplementary** **Table S1 A list of sequences for shRNAs and qRT-PCR primers**

| **Oligonucleotides for shRNA** | | | | |
| --- | --- | --- | --- | --- |
| *HDAC5*  shRNA-1 | | F: CCGGGCTAGAGAAAGTCATCGAGATCTCGAGATCTCGATGACTTTCTCTAGCTTTTTG  R: AATTCAAAAAGCTAGAGAAAGTCATCGAGATCTCGAGATCTCGATGACTTTCTCTAGC | | |
| *HDAC5*  shRNA-5 | | F: CCGGTCGGGAACCATCCTTGGAAATCTCGAGATTTCCAAGGATGGTTCCCGATTTTTG  R: AATTCAAAAATCGGGAACCATCCTTGGAAATCTCGAGATTTCCAAGGATGGTTCCCGA | | |
| **Primers sequences for qRT-PCR** | | | | |
| 1 | *HDAC5* | | F: ATGTCAGGTCGGGAACCAT | R: CGGCTTCACCTCCACTGT |
| 2 | *GAPDH* | | F: TTCTTTATGCTGGAGCCGAG | R: GAAACCTCTGGTCACAGTCG |
| 3 | *HDAC1* | | F: TTGTCTACTGGTGGTTCTGTGG | R: AGATGCCTCGGACTTCTTTG |
| 4 | *HDAC2* | | F: CAGATCGTGTAATGACGGTATCA | R: CCTTTTCCAGCACCAATATCC |
| 5 | *HDAC3* | | F: TGCATTGTGCTCCAGTGTG | R: CTTGACATATTCAACGCATTCC |
| 6 | *HDAC8* | | F: CCAAGAGGGCGATGATGATC | R: GTGGCTGGGCAGTCATAACC |
| 7 | *HDAC4* | | F: AGATCCTCATCGTGGACTGG | R: ATCGTCGTAGCGGTGGAG |
| 8 | *HDAC7* | | F: CACCTGCCTCACTGTCAGC | R: CGAGTCATAGATCAGCCCTGT |
| 9 | *HDAC9* | | F: AACATTACCTTGGGGCTTCC | R: CTGCGTCTCACACTTCTGCT |
| 10 | *A1CF* | | F: CCTATGCAGCAATTCCCAGT | R: ACAGAAGGGGCTCGGATAAT |
| 11 | *ATRX* | | F: TTGGTCGAAAGGAGTTGTCC | R: AAGGCTCTGGGTGACAAATG |
| 12 | *BAZ2B* | | F: GACCTCCAAATGTTGGCAAT | R: AGCCTGTTCCTGCTTTTGAA |
| 13 | *CBX7* | | F: GTCATGGCCTACGAGGAGAA | R: CTTGGGTTTCGGACCTCTCT |
| 14 | *CHD1L* | | F: CCTTGTGGAGGGATCTACCA | R: GCCTCTTCCTTTTGCCTCTT |
| 15 | *CHD2* | | F: AGGGGGAATCGAGTGCTTAT | R: TTCGGATTTCTCCCTTGATG |
| 16 | *EYA1* | | F: CACCAGCCAAGCAGTTACAG | R: ATCTGAACCTCGACGCAATC |
| 17 | *EZH1* | | F: TGTCGCTGTAAGACCCAGTG | R: AGGTCAGGGTCACATTCTCG |
| 18 | *ING4* | | F: GGAGAAGAAAGCTGCTCGTG | R: CTTCTTCTGGGCAGTCTTGG |
| 19 | *MBD5* | | F: ACGCACTTCATTTTCCATCC | R: GCTGTTGATTCACTGGCAAA |
| 20 | *MST1* | | F: CCAAGGTCTGGACGACAACT | R: AGAACTCTCGCTCGATCTGC |
| 21 | *NSUN6* | | F: ATGGTGGAGGACACAGAAGG | R: CAGGGTGCATCCAGAAGAAT |
| 22 | *PHF21A* | | F: ATCCAAAGCAAGAGGCAAGA | R: GGCTCAAAGACTGCTCCACT |
| 23 | *TET1* | | F: TCTGTTGTTGTGCCTCTGGA | R: GAAGCCCAAAGTTTTAAAGGC |
| 24 | *TLE4* | | F: GAGGTCAGTCCCATCTTCCA | R: CTTCTCAGCACCTCGGAAAC |
| 25 | *IGF-1R* | | F: CCAAAACTGAAGCCGAGAAG | R: TCCGCTTCCTTTCAGGTCT |
| 26 | *FOXA1* | | F: AGGGCTGGATGGTTGTATTG | R: ACCGGGACGGAGGAGTAG |

**Supplementary Figures and the Legends**


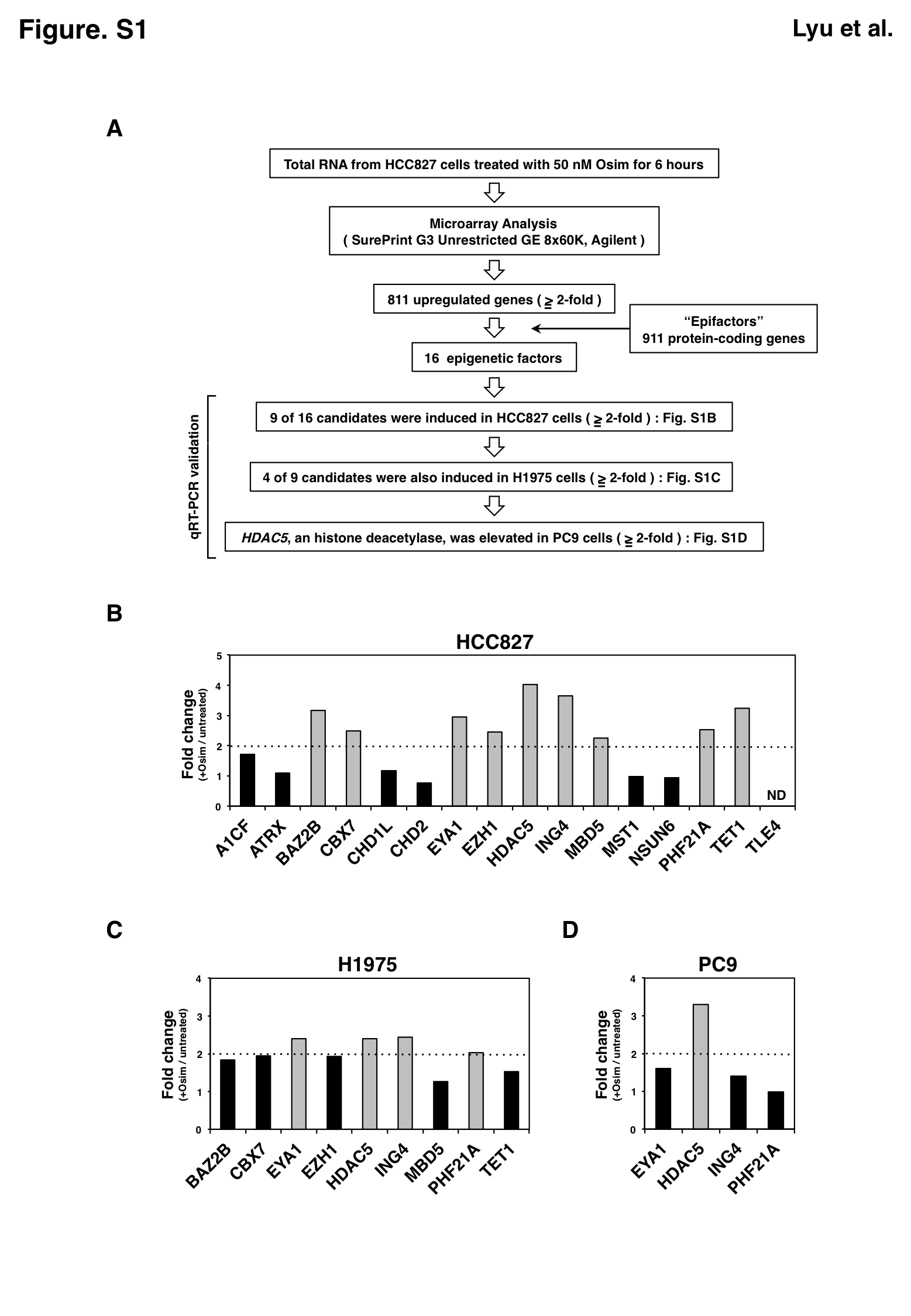


**Supplementary Fig. S1.** Microarray analysis was conducted to identify novel epigenetic factors induced by osimertinib. (A) Workflow for screening candidate epigenetic factors associated with the early response to osimertinib. Microarray data identified 16 candidates that were upregulated by more than 2-fold in osimertinib-treated HCC827 cells. Subsequently, qRT-PCR validation was performed to identify candidates (gray bars) showing a more than 2-fold increase (dot line) in the expression upon osimertinib treatment in HCC827 (B), H1975 (C), and PC9 cells (D).


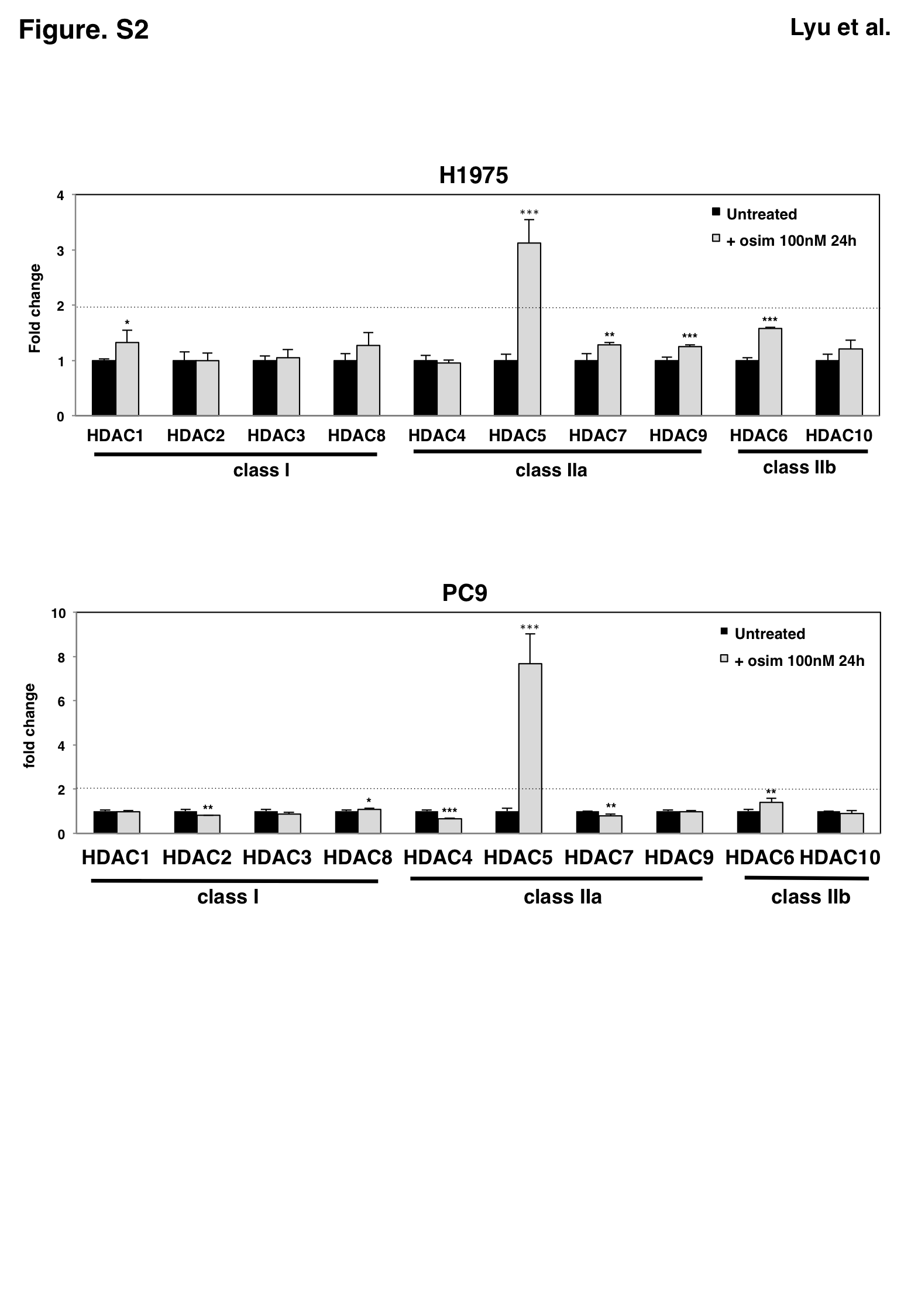


**Supplementary Fig. S2.** The expression patterns of HDACs belonging to class I and II families were shown in H1975 (top) and PC9 (bottom) cells treated with 100 nM osimertinib for 24 hours. Note that only *HDAC5* expression is more than 2-fold higher (dot line) in the treated cells. ****P* < 0.00.1, ***P* < 0.01, and **P* < 0.05, as analyzed by Student’s t-test.

**
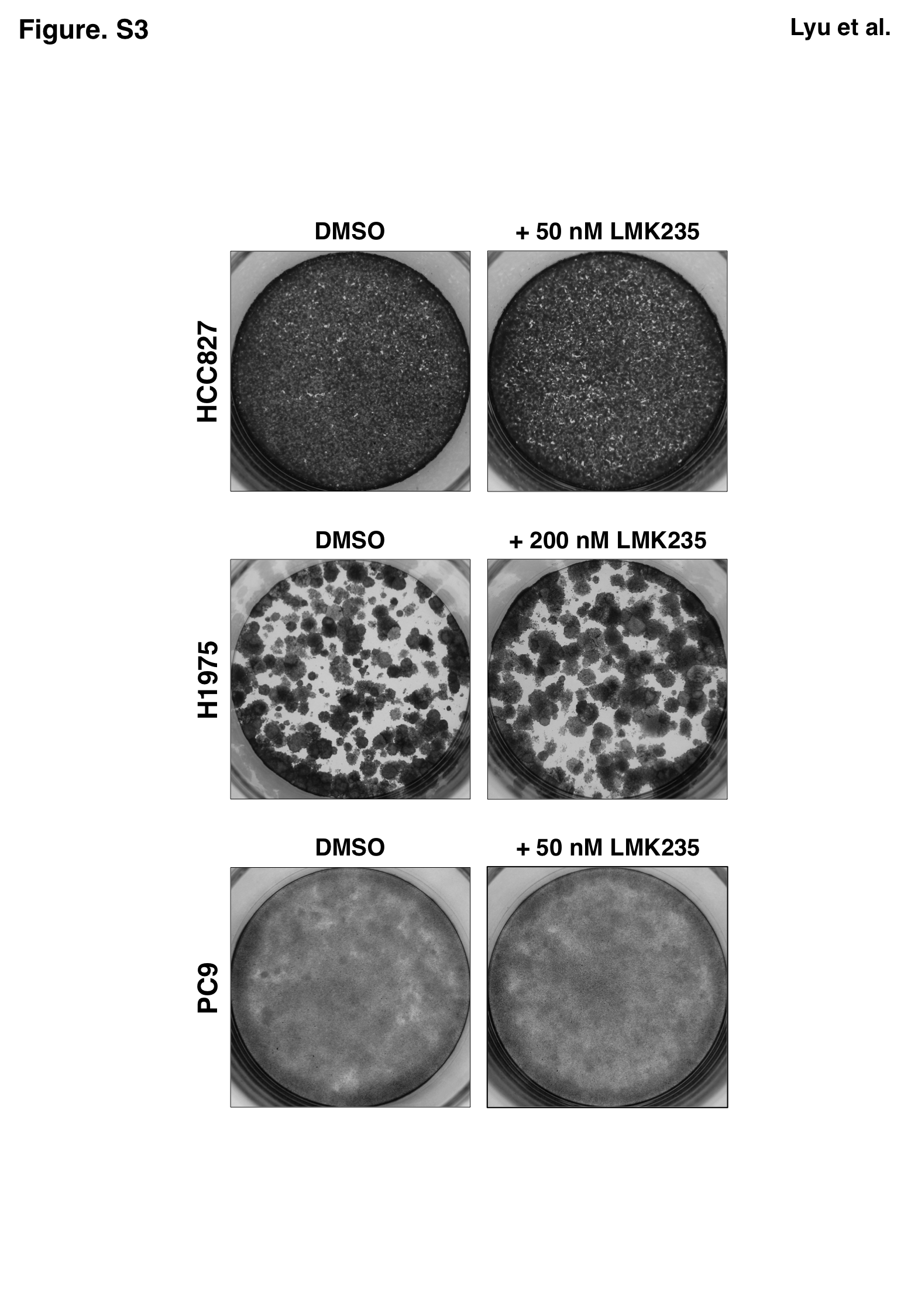
**

**Supplementary Fig. S3.** The representative images were presented for crystal violet staining with HCC827 (top), H1975 (middle), and PC9 cells (bottom) treated with LMK235 alone for 7 day, 12 days, and 9 days, respectively.

**
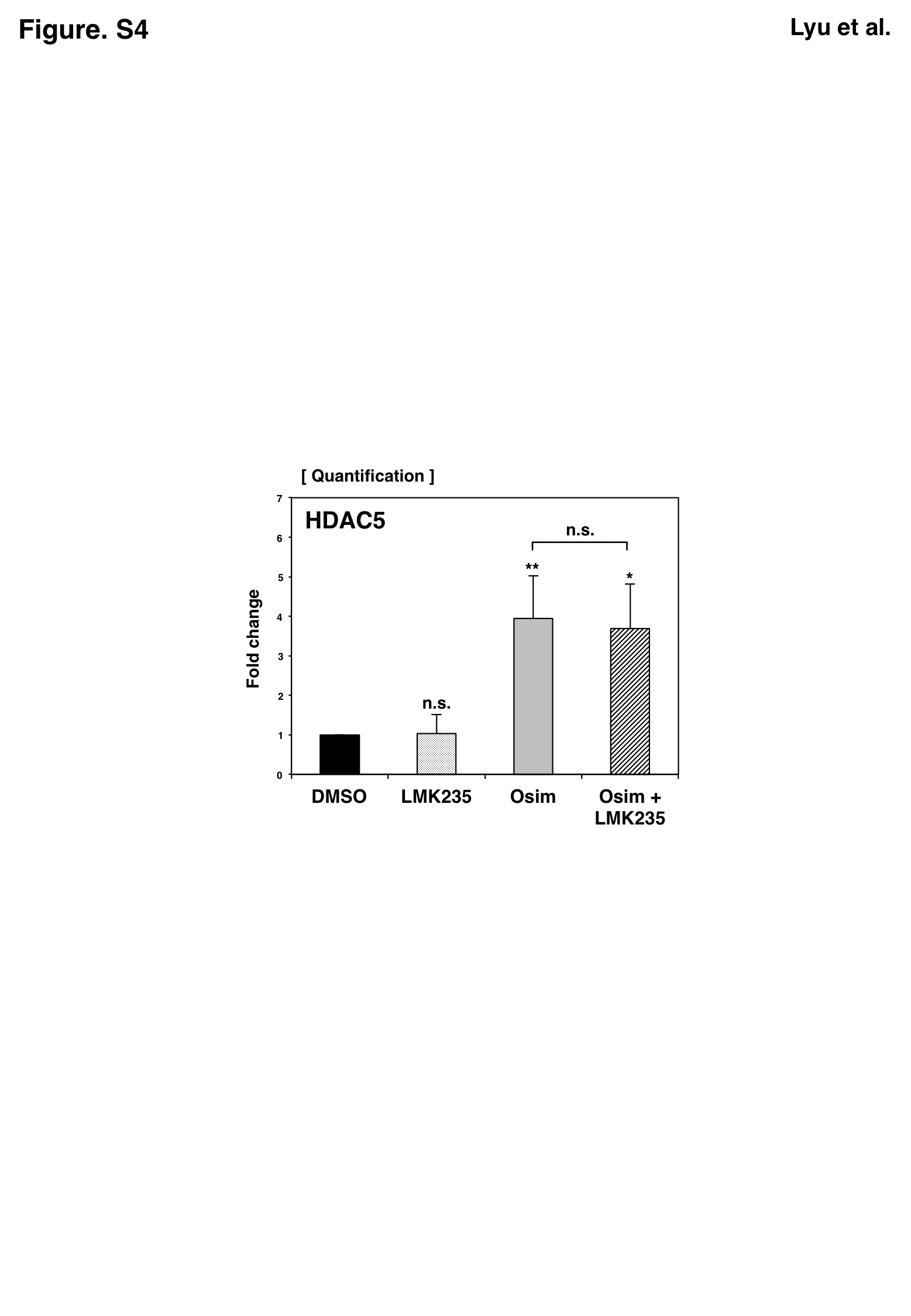
**

**Supplementary Fig. S4.** Quantification analysis of HDAC5 bands, as shown in Figure 4A, was performed in HCC827 cells treated with DMSO, LMK235, osimertinib, or their combination. ***P* < 0.01 and **P* < 0.05, as analyzed by Student’s t-test. n.s., not significant.

**
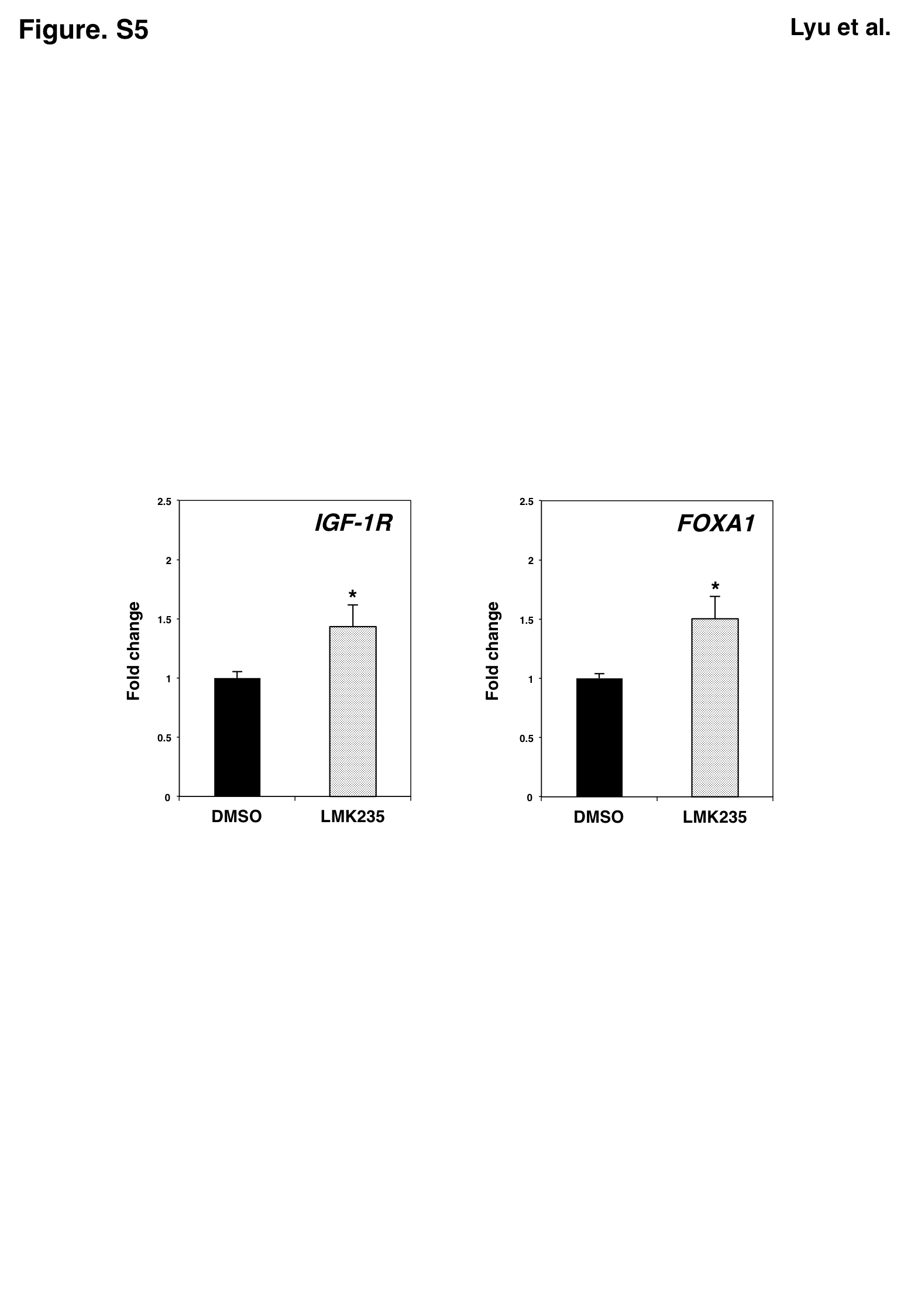
**

**Supplementary Fig. S5.** qRT-PCR analysis of *IGF-1R* and *FOXA1* in HCC827 cells treated with 50 nM LMK235. The experimental condition was the same as that of the experiment in Figure 4A. **P* < 0.05, as analyzed by Student’s t-test.
